# Optimization of Albuterol Delivery via Anesthesia Bag in Pediatric Critical Care

**DOI:** 10.3390/pharmaceutics17020218

**Published:** 2025-02-08

**Authors:** Sébastien Tessier, Victoria K. Ploszay, Christian Robitaille, Jigneshkumar Vaghasiya, Andrew J. Halayko, Louise Chartrand

**Affiliations:** 1 Children’s Hospital Research Institute of Manitoba, University of Manitoba, Winnipeg, MB R3E 3P4, Canada; seb_tessier@icloud.com; 2 St. Boniface Hospital, Winnipeg, MB R2H 2A6, Canada; ploszayv@gmail.com; 3 Department of Sociology, Liverpool Hope University, Liverpool L69 7ZX, UK; robitac@hope.ac.uk; 4 Department of Physiology and Pathophysiology, University of Manitoba, Winnipeg, MB R3E 3P4, Canada; vaghasij@myumanitoba.ca (J.V.); andrew.halayko@umanitoba.ca (A.J.H.); 5 Department of Internal Medicine, Max Rady College of Medicine, University of Manitoba, Winnipeg, MB R3E 3P4, Canada; 6 Biology of Breathing Group, Children’s Hospital Research Institute of Manitoba, Winnipeg, MB R3E 3P4, Canada; 7 Department of Respiratory Therapy, College of Rehabilitation Sciences, University of Manitoba, Winnipeg, MB R3E 0W2, Canada

**Keywords:** nebulization, mechanical ventilation, manual ventilation, pediatric aerosol

## Abstract

**Background/Objectives:** Aerosolized medications are common practice for mechanically ventilated pediatric patients. Infants often receive nebulized medications via hand ventilation using an anesthesia bag, but evidence on optimal aerosol delivery with this method is limited. For this study, various configurations of the Mapleson breathing circuit were tested to optimize albuterol delivery to a simulated pediatric model. **Methods:** Using a simulated pediatric lung model (ASL 5000) with the semi-open Mapleson anesthesia circuit, 2.5 mg/3 mL of albuterol sulfate solution was nebulized to a viral/bacterial filter (Respiguard 202). Four models were compared with varying fresh gas flows (FGFs), small-volume nebulizer (SVN) placements, and adjusting dead space. Five Registered Respiratory Therapists (RRTs) bagged the aerosol into a collection filter following defined ventilation parameters. Each model was tested in random order to avoid fatigue bias. Albuterol concentrations eluted from in-line filters were measured by spectrophotometry (absorbance at 276 nm). **Results:** No inter-user variability was observed among the RRTs. Significant differences in albuterol recovered were noted between models (One Way ANOVA, Tukey’s post hoc, n = 5). Model 4, with the nebulizer closest to the collecting filter, recovered 21.77 ± 1.89% of albuterol. The standard clinical model was the least effective, with only 0.10 ± 0.17% albuterol recovery. **Conclusions:** Modifying the anesthesia breathing circuit significantly improved aerosol drug delivery efficiency. Our findings suggest that current clinical practices for nebulized drug delivery are inefficient and can be markedly improved with simple adjustments in nebulizer positioning and gas flow within the circuit.

## 1. Introduction

Medications treating respiratory pathophysiology, especially in the pediatric population, are often nebulized and administered via inhalation [1,2]. This route of administration is preferred because it improves targeted delivery to the lungs and limits the systemic side effects of the drugs being delivered [3,4]. Commonly used inhaled therapies include aerosol drug formulations for short and long-acting bronchodilators, corticosteroids, mucoactive agents, antibiotics, and anti-viral medications [5]. Beyond conventional treatments, novel approaches such as inhaled stem cell therapy are gaining traction for their potential to heal damaged or immature lungs [6]. These advancements highlight the importance of developing a precise and efficient aerosolized drug delivery system, tailored to the specific needs of this vulnerable patient population.

The mechanics behind delivering aerosolized medications to spontaneously breathing patients are well established. Devices such as metered-dose inhalers (MDIs), dry powder inhalers (DPIs), and small-volume nebulizers (SVNs) are commonly employed due to their ability to deliver consistent doses of medication with minimal side effects [3,7]. These methods are effective when patients can coordinate deep, synchronized breaths with the device. However, critically ill patients, including intubated neonates and pediatric patients requiring mechanical ventilation, present a unique set of challenges for drug delivery. 

Mechanical ventilation is often used in critical care for ill patients requiring positive pressure support and provides consistent pressure, volume, rates, and the ability to synchronize aerosol delivery with each breath cycle, minimizing drug waste [3,4]. The efficacy of inhaled drug delivery during mechanical ventilation is determined based on the percentage of the loading dose delivered and where drug deposition occurs within the lung [2,5,7]. Drug deposition is also highly dependent on the physical, pharmacokinetic, and pharmacodynamic properties of the aerosol [8]. Several key factors impact drug delivery in the clinical environment: (1) ventilator settings; (2) configuration of the breathing circuit; (3) delivery device; (4) the medication; and (5) the patient [9,10].

Studies have demonstrated that for intubated patients’, aerosol drug deposition during mechanical ventilation is reduced with controlled positive pressure. Synchronization with spontaneously breathing patients not only improves drug delivery efficiency but also reduces the ventilator asynchronies where the patient and device are not working together [11]. The benefit of a spontaneous negative pressure breath from the patient allows for deeper medication deposition, improving ventilation to areas where there is perfusion and allows for better hemodynamic stability [12]. 

The effectiveness of manual ventilation in delivering aerosolized medications remains poorly understood, and to this day, significant gaps exist in the literature. Factors such as the impact of flow, the amount of dead space, and nebulizer placement can significantly influence aerosol deposition [13,14]. Unlike mechanical ventilation, which commonly uses a double-limb circuit, manual ventilation relies on a single-limb circuit, introducing additional variables that may affect drug delivery with inspiratory and expiratory flows passing through the same circuit. Variables such as the inspiratory flow, aerosol particle size, and the experience of the clinician delivering the medication all play a role [15].

The implications of these differences are important, as suboptimal drug delivery can lead to minimal therapeutic improvements, prolonged hospital stays, and increased healthcare costs. Furthermore, the lack of standardized protocols for manual ventilation raises concerns about variability in clinical practice, particularly in settings where it is a common practice. Studies have explored the impact of ventilator settings on aerosol deposition during mechanical ventilation, but there is a lack of research evaluating these factors in the context of manual ventilation using an anesthesia bag in the pediatric population [16].

Addressing these gaps is essential for optimizing patient care. For instance, neonates and infants have smaller airways, shorter time constants, complex respiratory mechanics and lower tidal volumes [17]. Understanding the impact of the circuit configuration, nebulizer placement, and gas flow dynamics is critical for developing evidence-based guidelines that ensure consistent and effective drug delivery.

This study aims to systematically investigate the impact of key variables—circuit configuration, nebulizer placement, and fresh gas flow on aerosol deposition during manual ventilation using an anesthesia bag. By employing a rigorous experimental design, this research seeks to provide actionable insights that can inform clinical practice and improve patient care. The findings are expected to fill a critical knowledge gap in respiratory medicine, particularly for clinicians who routinely use manual ventilation for aerosol delivery in their clinical practice. This work contributes to the broader field of aerosol therapy by highlighting the importance of optimizing drug delivery methods for critically ill pediatric patients.

## 2. Materials and Methods

### 2.1. Study Design

Four models of a single-limb breathing circuit were tested to evaluate the deposition of albuterol sulfate (USA Adopted Name for Salbutamol) using an in vitro design. Variables that impact deposition were controlled and kept constant for the four models, such as the type of nebulizer, the amount of albuterol, the simulator, and the collection filter. 

### 2.2. Nebulizer and Drug Delivery

This study used a jet-style SVN 8900 Series Nebulizer (Salter Labs, Vista, CA, USA) to deliver an albuterol sulfate solution to a collection filter (Respiguard 202, BOMImed, Winnipeg, MB, Canada) [7,18]. 

Aerosol particle size was not directly measured; the nebulizer and albuterol formulation are validated for clinical use and for pulmonary delivery. Specifically, aerosols are most effective when particle sizes range between 1 and 5 microns, facilitating deposition in the lower respiratory tract [2]. The nebulizer employed in our study has been demonstrated to produce aerosol particles within this optimal size range, ensuring effective delivery to the target sites in the lungs [8]. It is well reported that the choice of nebulizer and operating conditions significantly influence the mass median aerodynamic diameter (MMAD) of the aerosolized particles [18]. 

Given this prior validation, the study focused on evaluating the efficiency of different delivery models and the impact on albuterol deposition, operating under the premise that the particle size generated was within the therapeutically effective range. This approach allowed us to concentrate on optimizing delivery configurations to enhance drug deposition efficiency. With these properties already well established, the methods employed by clinicians in patient care settings remain the biggest impact factor for aerosol drug delivery. 

For in vitro testing, a high-fidelity lung simulator (ASL 5000, IngMar Medical, Pittsburgh, PA, USA) was configured to simulate the respiratory mechanics of a passive pediatric lung, excluding any spontaneous breathing efforts. The collection filter was placed proximal to the simulator, between the circuit and the lung model.

### 2.3. Drug Characteristics

Albuterol sulfate at a concentration of 2.5 mg/3 mL was used for each intervention. For operational requirements of the nebulizer, an additional 3 mL of sterile water was added to the solution. This is the standard dose for pediatric patients, diluted for the operational requirements of the SVN. The short-acting β_2_-agonist (SABA) is a common treatment of reversible restrictive lung diseases administered via inhalation but can also be administered orally, subcutaneously, intramuscularly, and intravenously [19]. Inhaled albuterol allows for a more effective and targeted treatment with a lower administered dose, minimizes systemic levels and side effects to other body systems by the medication, which are not the target of treatment [19]. For these reasons, an effective inhaled delivery model is essential. Administration via inhalation poses the greatest challenge in delivering albuterol and is the focus of this study.

### 2.4. Breathing Circuit Models

**Figure 1:**
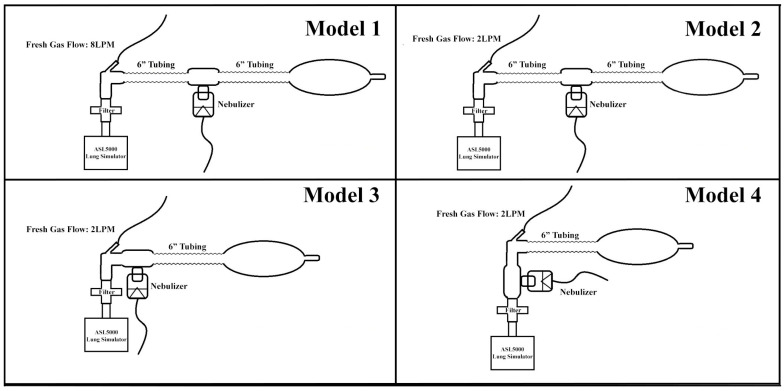
Model 1: FGF 8 LPM with nebulizer distal to FGF; Model 2: FGF 2 LPM with nebulizer distal to FGF; Model 3: FGF 2 LPM with nebulizer proximal to FGF; Model 4: FGF 2L PM with nebulizer between FGF and filter.

Four configurations (described below, see Figure 1) of a Mapleson anesthesia breathing circuit with a 500 mL reserve bag, which is commonly used to support ventilated pediatric patients and deliver aerosol medication at the facility where the study took place (Mercury Medical, Clearwater, FL, USA).

Figure 1 illustrates all four models. Model 1 served as the control configuration because it reflects the practice within our pediatric facility. In Model 1, the total flow of oxygen within the circuit is up to 16 liters per minute (LPM) (8 LPM of FGF plus 8 LPM to run the nebulizer). For Model 2, the flow coming from the jet nebulizer (8 LPM) could not be decreased as it would impact the performance of the nebulizer. To decrease total flow, the FGF was decreased to 2 LPM so that the total flow within the circuit would amount to 10 LPM. This total flow for the Mapleson breathing circuit allows the RT to properly manage ventilation. In instances where aerosolized medications are not being delivered, this is the flow used to properly operate the bag. Flow rates for Model 3 were unchanged from Model 2 but eliminated dead space by removing the 6″ limb between the nebulizer and FGF. Finally, Model 4 employs the same circuit components as Model 3 but relocates the nebulizer between the FGF and the filter, positioning it proximally to the collection filter.

### 2.5. Ventilation Parameters

Five pediatric Registered Respiratory Therapists (RRTs) conducted the intervention and delivered the aerosol into the collection filter. Each intervention had a duration of 10 min. The RRTs were asked to target the following ventilation parameters for each model: Respiratory Rate 40/min, Peak Inspiratory Pressure 25 cmH_2_O, PEEP 10 cmH_2_O. The breathing circuit included a pressure manometer, which provided the RRTs with pressure feedback during each intervention, as is reflective of the practice where the study was conducted. However, the RRTs were blinded to the tidal volume and respiratory rate that they were producing during hand ventilation. The respiratory rate target is based on clinically accepted parameters for this population. Each RRT tested each of the four models once and in random order to avoid fatigue bias (a total of 20 interventions). 

### 2.6. Drug Deposition and Measurement

Following each intervention, the collection filters were eluted with 10 mL of 0.1 mM HCl. The filters were covered with a parafilm (Bemis, Neenah, WI, USA) shaken for a total of three minutes to extract the deposited albuterol. The sample was aspirated with a clean pipette and placed in a quartz cuvette for measurement. The concentration of albuterol sulfate was then measured using calibrated spectrophotometry (Jasco CAF-110, Jasco, Easton, MD, USA) with absorbance set at 276 nm. The measurements were verified against a standard curve that was established using the same control settings and device [13,18,20].

### 2.7. Statistical Analysis

The relationship between each RRT for each model was analyzed by Pearson’s correlation. The models were compared for statistical significance using one-way ANOVA with Tukey’s multiple-comparison test. Analysis was performed using GraphPad Prism version 10.1.1 (GraphPad, San Diego, CA, USA) with a significant threshold of *p* < 0.05.

## 3. Results

### 3.1. Comparison of Albuterol Deposition Across Therapist

Using Pearson’s correlation analysis, we found no statistically significant difference in the effectiveness of hand ventilation by respiratory therapists across all aerosol models tested (Table 1). This suggests consistency in the delivery technique among therapists, regardless of the specific model used.

### 3.2. Comparison of Albuterol Deposition Across Models

**Figure 2:**
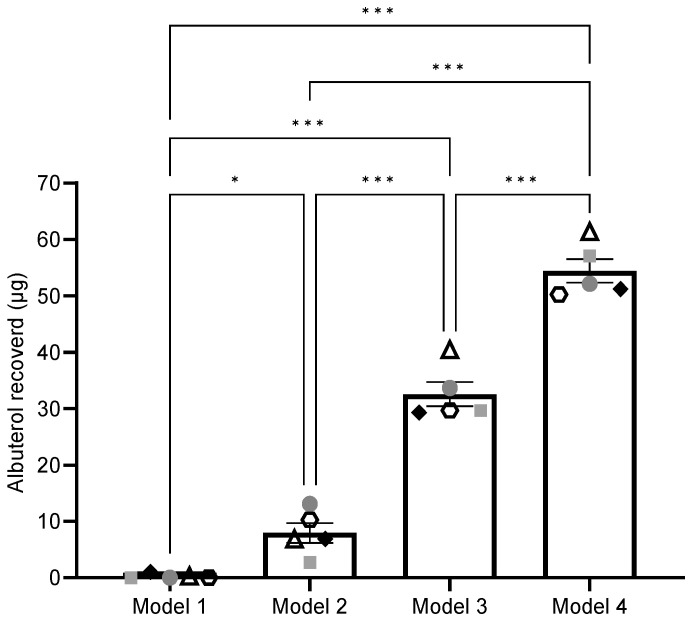
Comparison of albuterol recovered from each delivery model. Each data point represents results from individual participant (n = 5, each user assigned a unique symbol). Data were analyzed by one-way ANOVA with Tukey’s multiple-comparison test using GraphPad Prism version 10.1.1 (San Diego, CA, USA). Normality of residuals passed Shapiro-Wilk test. Data represent mean ± SE, *p* < 0.05 was considered significant, * *p* < 0.05, *** *p* < 0.001.

Albuterol deposition for all four models was compared using a one-way ANOVA followed by Tukey’s multiple-comparisons test (Table 2, Figure 2). The results demonstrated significant differences in albuterol recovery and efficiency among the models.

Model 1 (Control): Model 1, representing the control configuration, was the least efficient. It recovered 0.27 ± 0.44 µg of albuterol, equating to 0.10 ± 0.17% of the total nebulized dose.

Model 2: Model 2 showed a significant improvement in albuterol recovery, with 7.97 ± 3.92 µg recovered, corresponding to an efficiency of 3.20 ± 1.54%. This was significantly higher compared to Model 1 (*p* < 0.05).

Model 3: Removing dead space in Model 3 further improved albuterol deposition. The recovery increased to 32.60 ± 4.75 µg, equating to an efficiency of 13.03 ± 1.91%. Model 3 demonstrated significant improvement compared to both Model 1 (*p* < 0.001) and Model 2 (*p* < 0.001).

Model 4: Model 4 was the most efficient configuration, with 54.4 ± 4.70 µg of albuterol recovered, achieving an efficiency of 21.77 ± 1.89%. Model 4 exhibited significantly higher recovery and efficiency compared to all other models (Model 1, Model 2, and Model 3, *p* < 0.001). 

## 4. Discussion

Aerosol deposition was significantly increased with subsequent circuit models. Specifically, each of the following had an impact on the aerosol delivery: (1) total gas flow within the circuit; (2) total dead space in the circuit; and (3) placement of the nebulizer relative to the fresh gas source and filter. Modifying each of these technical components had significant impact on drug delivery. 

The Mapleson breathing circuit requires a constant flow of gas for positive pressure ventilation. The gas flow fills the circuit and the collapsible bag with a reserve of fresh oxygen to generate pressure within the breathing circuit, with the pressure regulated using the terminal outlet. The proximity of fresh gas supports manual ventilation by helping evacuate and purge the circuit of the patients’ exhaled gases. However, gas flow, also known as bias flow or flow, inversely affects aerosol deposition in a variety of settings, including High Flow Nasal Cannula and Invasive Mechanical Ventilation [8,14]. Ari et al. [8] reported that higher gas flows decrease drug delivery as it adds a dilutive effect independent of the nebulizer position. The trend sees a decrease in drugs delivered with higher flows in the breathing circuit. In practice, the operational flow for the Mapleson breathing circuit is set between 8 and 10 LPM. Adding the nebulizer to the circuit requires an additional 8 LPM, which nearly doubles the total flow. With combined flows of up to 18 LPM in our control (Model 1), it is likely that this contributed significantly to the dilution of aerosolized albuterol and thus diminished its deposition in a simulated lung [4]. For Model 2, the decrease in flow does significantly increase the deposition of aerosolized albuterol when significance is set at *p* < 0.05.

Another concern with the higher flow is that decreased aerosol deposition can result from the effect of turbulent airflow, causing inertial impaction with the walls of the circuit [21]. Furthermore, in a single-limb configuration, the flow of gas is bidirectional within the circuit when the expiration opening is distal to the patient. In a static state, when no positive pressure breaths are being delivered by the provider, the only escape for gas within the circuit is distal to the patient, and the flow of gas is directed towards the opening. These problems are exacerbated by the distance between the nebulizer and the patient’s airway, in that more volume (and possible turbulence) within the circuit needs to be overcome before the aerosol reaches its intended destination, or in this instance, the collection filter at endotracheal tube (ETT). Thus, the volume in the circuit between the patient and the nebulizer is equivalent to dead space. When the nebulizer is distal to the FGF, an estimated 133 mL of oxygen enters the circuit for every breath (every second) when the gas flow is set at 8 LPM. With a typical inspiratory time for this pediatric population (0.7 s), 93 mL of fresh gas would flow into the circuit. In addition, the volume of the circuit between the nebulizer and FGF is about 58 mL. To overcome the presumed dead space, a breath would need to be above 151 mL (6′ tubing = 58 mL + 93 mL of gas that enters the circuit = 151 mL) in volume before the aerosol would reach the ETT of an intubated patient. Since such volumes are inappropriate for a 10 kg infant with a normal typical tidal volume of 70 mL (7 mL/kg), lower quantities of medication will be deposited. Most albuterol travels toward the exit of the circuit without the opportunity to reach the patient or, as our study demonstrates, with little collected by the filter. This is a substantial finding as the control reflects current use in clinical practice and recovering only 0.10% of the loading dose. With the single-limb circuit, the control models’ dead space is significant, and FGF is too high to deliver any medication. During the interventions, you could, in fact, see the generated aerosol enter the circuit and immediately be directed towards the bag opening rather than fill the spaces between the collection filter and nebulizer.

To assess the impacts of dead space on aerosol deposition, the 6″ piece of corrugated tubing between the nebulizer and fresh gas source was removed (Model 3). In clinical practice, it was thought that this part of the circuit acted as a reservoir for aerosolized medication, similar to a Metered Dose Inhaler with a spacer [15]. As described above, the intervention saw no amount of aerosol remain in suspense within the piece of corrugated tubing. The 6-inch, 22 mm diameter tube constitutes a volume of approximately 58 mL (57.93 mL). Decreasing airflow to 2 LPM results in 33 mL of oxygen to entering the circuit as a fresh gas source every second (21 mL, with the same inspiratory time of 0.7 s). Equally, removing the 6″ tubing between the nebulizer removes the dead space. In this instance, the component did not provide any clinical benefit to medication delivery. The aerosolized medication only needs to overcome the 21 mL FGF entering the circuit, which is easily attained with a tidal volume of 70 mL. These changes to the circuit were positively reflected in our results; with the FGF of 2 LPM, the total flow within the circuit was clinically manageable at 10 LPM. This is the ideal flow for ventilating a patient, and in this scenario, the mean medication deposition was 13.03 ± 1.91%, which is substantially higher than that attained with the configurations for Model 1 and Model 2.

During mechanical ventilation with a dual limb circuit, the amount of medication delivered to a collection filter is highest when the nebulizer is closest to the ventilator or further away from the patient’s airway [2,10]. However, for single-limb circuits ventilating manually by hand, the deposition is highest when the nebulizer is most proximal to the patient’s airway [2]. In our test of the Mapleson breathing circuit, the model that had the highest deposition of aerosolized medication occurred when the nebulizer was placed between the FGF source and the collection filter, in addition to decreasing the FGF and removing dead space in the circuit. Model 4 also used the full expiratory limb and bag as a reservoir for the bias flow, which is an important consideration for circuit design. Between the nebulizer and opening at the distal end of the bag, aerosol fills the space continuously between breaths. This was equally observed during the intervention. In the other models, the additional 6″ circuit placed between the nebulizer and the patient stayed visibly empty, negatively impacting delivery. Finally, the FGF in Model 4 circulates the medication within the nebulizer T-piece towards the airway opening; the entire circuit would arguably now serve as a reservoir. 

As part of this project, the potential for inter-user variability was assessed. For this intervention, there were no significant differences in aerosol deposition between RRTs for each model tested. The clinical experience of the RRTs did vary between 3 years’ experience to over 25 years’ experience. With no inter-user variability, there is limited benefit in expanding on the impact of each participating clinician. What is worth denoting is that the RRTs have the potential to significantly impact deposition based on how they manage the frequency, pressures, and timing of the breaths delivered. 

The clinical implications of these findings are substantial. The Mapleson breathing circuit, or variations in this model, are widely used in pediatric anesthesia and critical care. The clinician reported benefits of hand ventilation lie in the ability to continuously control, adjust, and monitor ventilatory pressures. The target population for this study is unable to benefit from receiving aerosolized medication with the available mechanical ventilators due to the presence of a proximal hot wire anemometer flow sensor. The diameter of the inner flow sensor is less than 3 mm and has mesh wiring at each end to protect the hot wires from patient secretions. These two components significantly hinder the potential for effectively delivering aerosol. In addition, vendor specifications advise against introducing a mist or aerosol as it may damage the flow sensor device and impact ventilator performance. 

Our results suggest that without optimizing flow rates, dead space, and nebulizer placement, the current configuration (Model 1) recovers only 0.10% of the nebulized dose, leading to subtherapeutic drug delivery and poorer clinical outcomes. Conversely, the modifications tested in Model 4 increased deposition at the airway opening by over 200-fold, demonstrating the potential for significant therapeutic improvement given the limited options available within this facility.

Implementing these modifications could improve medication delivery for intubated pediatric patients, reducing treatment failures and associated healthcare costs. Furthermore, the simplicity of these adjustments, such as reducing dead space and repositioning the nebulizer, makes them feasible for widespread adoption in circumstances where it is not possible to leverage a ventilator to deliver the medication.

## 5. Conclusions

The effectiveness of aerosol delivery in pediatric populations using manual ventilation with a gas flow-dependent anesthesia circuit was tested in this study. Significant improvements in aerosol deposition were measured with circuit modifications, highlighting the importance of optimizing the technical components of the breathing circuit. Medications used to treat respiratory conditions in a clinical setting are readily assessed for any impact of their pharmacodynamic properties as an aerosol suspense delivered to the lung. Most nebulizers do not use heat or generate physical changes to the chemical component of the medication, which is why they continue to be the delivery method of choice over an alternate route of administration. 

It is recommended that further research be conducted to evaluate how these improvements translate into therapeutic effects and patient outcomes in real-world clinical settings. The delivery of aerosols to the endotracheal tube (ETT) of intubated pediatric patients was the primary focus of this study. It serves as a bridge by providing an important perspective beyond the pharmaceutical properties and establishing that clinical delivery can have an all-or-nothing impact. The characteristics of different ETT properties, such as diameter and length, were not addressed, although prior research has demonstrated that ETT size significantly impacts aerosol deposition [22]. Future investigations should consider incorporating these factors to better understand their influence on drug delivery.

A simulated lung model representing a homogenous pathology was used, which excluded patient-specific variations such as spontaneous respiratory efforts. This approach, while valuable to control parameters in respiratory care, introduces limitations in generalizing the findings to clinical scenarios where diverse pathophysiology is often present in pediatric critical care. It is recommended that future research incorporate dynamic and heterogeneous models to better reflect real-world conditions. With deposition significantly increased with the nebulizer placed proximal to the ETT, its potential impact on patient comfort and compliance should be further examined, especially in populations where proximity to the airway may pose challenges.

The use of a jet nebulizer was chosen because it reflects current clinical practice for delivering aerosolized medications to intubated pediatric patients. Although cost-effective and widely used, performance-wise, jet nebulizers are less efficient than newer technologies, such as vibrating mesh nebulizers (VMNs), which consistently deliver smaller particle sizes [8]. Comparative studies examining the relative deposition efficiency of VMN technologies with traditional devices such as the jet nebulizer are recommended, particularly in both manual and mechanical ventilation contexts. Additional research on aerosol particle size is strongly encouraged. While the particle size produced by the nebulizer used in this study has been previously validated as being within the optimal range for pulmonary delivery, direct measurements of aerosol properties, such as mass median aerodynamic diameter (MMAD), geometric standard deviation (GSD), and electrokinetic stability, could provide valuable insights into the mechanisms influencing drug deposition. These investigations would enhance the understanding of how particle size and aerosol characteristics interact with circuit configurations and flow dynamics to impact overall efficacy.

This research provides actionable evidence for optimizing aerosol therapy in pediatric populations using manual ventilation. It is suggested that clinicians (1) position the nebulizer proximal to the patient’s airway to minimize dead space; (2) remove unnecessary dead space within the circuit to reduce airflow disruption; and (3) limit total flow to minimize turbulence and enhance the operation of the manual ventilation bag. While these findings are promising, several variables remain unexplored. The characteristics of different medications, the comparative efficacy of mechanical versus manual ventilation in aerosol delivery, and the direct relationship between particle size and therapeutic outcomes require further investigation. Future studies focusing on these aspects are strongly recommended to provide evidence that can further improve the management of intubated pediatric patients.

## Figures and Tables

**Table 1:** Analysis of inter-user variability by Pearson’s correlation coefficient test (n = 5 participants for each model). For each models the difference between participants were not significant (ns).

Correlation Participants vs. Model	Model 1	Model 2	Model 3	Model 4
Pearson r	0.56	−0.64	0.44	0.65
95% CI	−0.64 to 0.97	−0.97 to 0.56	−0.72 to 0.95	−0.54 to 0.97
R^2^	0.31	0.41	0.19	0.43
P (two-tailed)	0.328	0.246	0.459	0.231
P Summary (alpha = 0.05)	ns	ns	ns	ns

**Table 2:** Tukey’s multiple comparison test for each model. * *p* < 0.05, *** *p* < 0.001.

Tukey’s Multiple Comparisons Test	Mean Diff.	95.00% of Diff.	Below Threshold?	Summary	Adjusted *p* Value
Model 1 vs. Model 2	−7.70	−14.7 to −0.681	Yes	*	0.029
Model 1 vs. Model 3	−32.3	−39.3 to −25.3	Yes	***	<0.001
Model 1 vs. Model 4	−54.2	−61.2 to −47.1	Yes	***	<0.001
Model 2 vs. Model 3	−24.6	−31.6 to −17.6	Yes	***	<0.001
Model 2 vs. Model 4	−46.4	−53.5 to −39.4	Yes	***	<0.001
Model 3 vs. Model 4	−21.8	−28.9 to −14.8	Yes	***	<0.001

## Data Availability

Data will be available upon request to the corresponding author.
